# Off-pump occlusion of trans-thoracic minimal invasive surgery (OPOTTMIS) on simple congenital heart diseases (ASD, VSD and PDA) attached consecutive 210 cases report: A single institute experience

**DOI:** 10.1186/1749-8090-6-48

**Published:** 2011-04-13

**Authors:** Qing-kui Guo, Zhi-qian Lu, Shao-fei Cheng, Yong Cao, Yong-hong Zhao, Cheng Zhang, Yue-li Zhang

**Affiliations:** 1 Department of Cardio-thoracic Surgery, Shanghai NO.6 People Hospital Affiliated Shanghai Jiao Tong University, NO. 600 Yishan Road, Shanghai, 86: 200233, China

**Keywords:** Off-pump, Occlusion, Minimal invasive surgery, Congenital Heart Disease, Trans-esophageal Echocardiography

## Abstract

**Objective:**

This paper intends to report our experiences by using an operation of off-pump occlusion of trans-thoracic minimal invasive surgery (OPOTTMIS) on the treatment of consecutive 210 patients with simple congenital heart diseases (CHD) including atrial septal defect (ASD), ventricular septal defect (VSD) and patent ductus arteriosus (PDA).

**Methods:**

The retrospective clinical data of OPOTTMIS in our institute were collected and compared to other therapeutic measures adopted in the relevant literatures. After operation, all the patients received electrocardiography (ECG) and echocardiography (echo) once a month within the initial 3 months, and no less than once every 3 ~ 6 months later.

**Results:**

The successful rate of the performed OPOTTMIS operation was 99.5%, the mortality and complication incidence within 72 hours were 0.5% and 4.8%, respectively. There were no major complications during peri-operation such as cardiac rupture, infective endocarditis, strokes, haemolysis and thrombosis. The post-operation follow-up outcomes by ECG and echo checks of 3 months to 5 years showed that there were no III° AVB, no obvious Occluder migration and device broken and no moderate cardiac valve regurgitation, except 1 VSD and 1 PDA with mild residual shunts, and 2 PDA with heart expansion after operation. However, all the patients' heart functions were in class I~II according to NYH standard.

**Conclusion:**

The OPOTTMIS is a safe, less complex, feasible and effective choice to selected simple CHD patients with some good advantages and favorable short term efficacies.

## Backgrounds

Congenital heart diseases (CHD) are common complaints with incidence of 8‰ ~ 12‰ in China, including atrial septal defect (ASD), ventricular septal defect (VSD) and patent ductus arteriosus (PDA). Approximately, there are 150,000 ~ 200,000 Chinese infants born with CHD every year [1]. Now days, there are different treatment methods to CHD as traditional open surgery, physician interventional occlusion through intravenous catheter delivery system, several minimal invasive surgery using various small incision, video assisted thoracoscope, robotic systems, hybrid approaches, etc. More or less, these methods have their shortcomings, such as, sever body injuries by extended open-chest incision and cardiopulmonary bypass (CPB), many morbidities and complications, long skin scars, demanding special apparatus and long learning time-cure to master the sophisticated procedures, and radiation damages to intervention physicians and patients that cannot be avoided. Once patients' venous vessels and inner cardiac structures were damaged by catheter and wire due to the long pathway and slender sheath and wire, then open surgery must be transferred for rescue [2-11]. In recent decades, hybrid approaches have been accepted by people gradually with the rapid development of minimal invasive techniques and equipments. As one technique of hybrid approach, OPOTTMIS has grown into a safe and effective treatment method for simple CHD [12-24]. In this article, we reported the experiences of consecutive 210 cases simple CHD patients treated with OPOTTMIS in our hospital during July 2005 ~ October 2010.

## Materials and methods

### 1. Patient information

The consecutive 210 simple CHD patients (96 males and 104 females) with 3 ~ 56 (18.92 ± 15.64) years of old and 8.0 ~ 54.5 (24.78 ± 16.63) kilograms of weight, were diagnosed through physical examination, chest X-Ray, ECG and echo including trans-thorax echocardiography (TTE) or/and trans-esophageal echocardiography (TEE). There were 92 cases of ASD with diameter of 21.5 ± 11.6 mm, 63 cases of VSD with diameter of 9.8 ± 3.2 mm, and 55 cases of PDA with diameter of 7.6 ± 1.8 mm (including 1 cases of adult PDA approach to severe pulmonary hypertension).

### 2. Preoperative preparation

The probable risk of OPTTMIS, anaesthesia, blood transfusion and transform to open surgery with CPB must be informed to the patients and their family members. All the patients were asked to sign the informed consent before operation to accept the treatment with OPOTTMIS method. Occluders and delivery systems, ultrasonograph (Mode: PHILIPS 4500) assembled with sterilized probes for intra-operation TTE and TEE checks, blood for transfusion, CPB machines and open operation pertinent equipments must be prepared for use when needed.

### 3. Occluders

**Figure 1:**
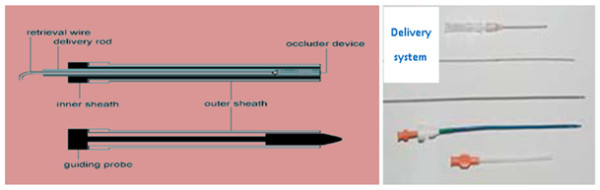
**Delivery systems and self-made devices used for OPOTTMIS**. Outer sheath; Inner sheath; Guiding probe; Delivery rod; Retrieval wire.

**Figure 2:**
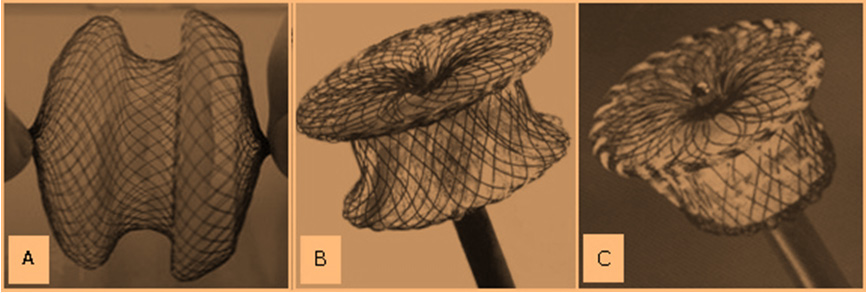
**ASD, VSD and PDA Occluders used in the OPOTTMIS**. (Made in Shanghai shape memory alloy material Ltd. Co., CN, No.: 20043770007). A: ASD Occluder; B: VSD Occluder; C: PDA Occluder. The Occluders are made from Nitinol materials.

The special double lumen equipment of delivery systems for OPOTTMIS are composed by the outer and inner sheath, delivery rod, retrieval wire, guide probe, and occlude device (Figure 1) [24], and the sheath diameter is Fr 6 ~ Fr 26. The sizes of ASD, VSD and PDA Occluders (Figure 2) are different from 15 ~ 46 mm, 8 ~ 22 mm and 6 ~ 16 mm, respectively. The experienced formulation of Occluder size selection for OPOTTMIS were shown (Table 1).

**Table 1:** Occluder size select for OPOTTMIS.

Disease	The experienced formulation
ASD	Y = X + 4 ~ 6 (mm)
VSD	Y = X + 4 ~ 6 (mm)
PDA	Y = X + 2 ~ 4 (mm)

Y: size of Occluder; X: max diameter of defect tested by UCG.

### 4. Inclusion and exclusion statements

Applications and contraindications of OPOTTMIS for simple CHD patients were accepted according to the ACC/AHA 2008 adult CHD administer guidelines [25] and shown as follows (Table 2, Table 3 and Table 4).

**Table 2:** Applications of OPOTTMIS for simple CHD.

Disease	OPOTTMIS applications
ASD	ASD upper margin ≥ 4 mm, inferior margin ≥ 5 mm, with the defect marginal space to the annulus of MV ≥ 5 mm; Atrial septum longitude > Occluder umbrella diameter within LA; ASD diameter < 38 mm; Secundum ostium with diplopore (one larger and the other smaller).
	
VSD	Peri-menbranous VSD; muscular VSD ≥ 4 mm; Inferior pulmonary trunk VSD with marginal space to the RCC ≥ 2 mm, without sever AV prolapse and regurgitation; Muscular VSD affecting cardiac hemodynamic.
	
PDA	Fistular PDA; Fenestrae PDA; Infundibular PDA; Left to right shunt PDA none malformation needing operation rectification; PDA diameter ≥ 4 mm.

LA, left atrial; RCC, right coronary cusp; AV, aortic valve.

**Table 3:** Contraindications of OPOTTMIS for simple CHD.

Disease	Respective contraindications	Common contraindications
ASD	Margin < 4 mm; Foramen primium defect with MV cleavage; Mesh shaped ASD; SVC, IVC and CS ASD.	Sever right to the left shunt; Eisenmenger syndrome; Atrial thrombus; Complex cardiac malformation; Uncontrolled pulmonary infection; Any pre-operation serious infective diseases within one month (as ABE or systemic infection); Malignant diseases with life expectancy < 3 years; Cannot get consent and signature.
		
VSD	Multiple small muscular VSD	
		
PDA	Dumbbell PDA; Combined with sever pulmonary calcification, inflammation, or hypertension.	

MV: mitral valve; ABE: acute bacterial endocarditis. SVC: superior vena cava; IVC: inferior vena cave; CS: coronary sinus.

**Table 4:** Complications of OPOTTMIS for simple CHD.

Stratification	OPOTTMIS complications
**Operation relative **(approximately 5%)	Occulder migration; Residual shunt; Bleeding; Arthythmia (Conduction block, Atrial fibrillation); Hemolysis; Blood thrombus; Air embolus; Infection; Hemopneumothorax; Pericardial tamponade; Death.
	
**TEE relative **(approximately 1 ~ 3%)	**Serious**: Death; Esophagus and gastric perforation; Upper gastrointestinal hemorrhage; Arthythmia; Aspiration pneumonitis. **Mild**: Temporary air duct compression; Ventilation restriction; Descending aorta compression.

TEE, trans-esophageal echocardiography.

### 5. The procedure of OPOTTMIS

(1) The patients were placed in the supine position and administered by inhaled general anesthesia through single or double lumen tracheal catheter intubation. The defect malformations were verified by the TEE checks through the probes placed into the patients' esophagus.

(2) As a general rule in most cases, the selected chest wall incisions of ASD, VSD and PDA were located at the third or fourth intercostal space right lateral sternal with 2.0 ~ 3.0 cm in length, distal midterm sternotomy to xiphoid with 3.0 ~ 5.0 cm in length, and the second intercostal space of left lateral sternal with 2.0 ~ 3.0 cm in length, while the selected cardiac puncture sites apart from coronary arteries were located at the right atrium wall, the right ventricular wall with tremor, and the primary pulmonary arterial wall with obvious thrill, respectively.

(3) Surgical procedures

①Patients were placed on the operation-table at prostrate position with the operation lateral body raised and sloped to 30° ~ 45° using cushions. Then the selected chest wall was cut by a scalpel and exposed with a small rib retractor. After pericardium incision and sling to the chest wall using gross silk suture, heparin was administrated to the patient by intravenous injection with dose of 0.5 ~ 1.0 mg/kg. When ACT (accelerated clotting time) surpassed 200 s, double purse-string suture or double U-shape suture were sewed at the site of the selected cardiac wall using 4/0 Prolene lines attached with double needles and small Teflon or pericardium pads.

②The outer self-made delivery sheath and guide probe were punctured into the appropriate cardiac or main pulmonary chamber through the central of the suture. After the guide probe pulled out and a guide wire put into the outer sheath promptly, the delivery rod (also named as the inner sheath) was pushed into the corresponding chamber of the heart along the guide wire through the defects under TEE surveillance.

③The chosen Occluder with right size was rinsed within 1% concentration of heparin normal saline solution for about 5 minutes. Then the guide wire was pulled out while the Occluder stitched with a safe wire on it was placed into the inner delivery sheath as soon as possible to prevent massive bleeding and air entering into the cardiac or main pulmonary chambers. Under the surveillance of ECG and TEE checks (TTE or trans-epicardium echo when needed), the "push-pull" test was performed to adjust the position of the Occluder release and ensure that its waist will straddle on the edges of the defects firmly and well, and there were no moderate to heavy residual shunts, no atrioventricular and semilunar valves influences, no III° AVB and no massive air in cardiac chambers. After that, the delivery sheath and the safe wire were cut off and pulled out of the heart, then the double purse-string or double U-shape suture with Prolene lines were ligated strictly after lungs inflation. Once the operating fields were inspected carefully and found no observed bleeding, the thoracic incisions were closed layer by layer. Normally, there was no the needs of blood transfusions and closed thoracic drainages but for the massive bleeding patients.

**Figure 3:**
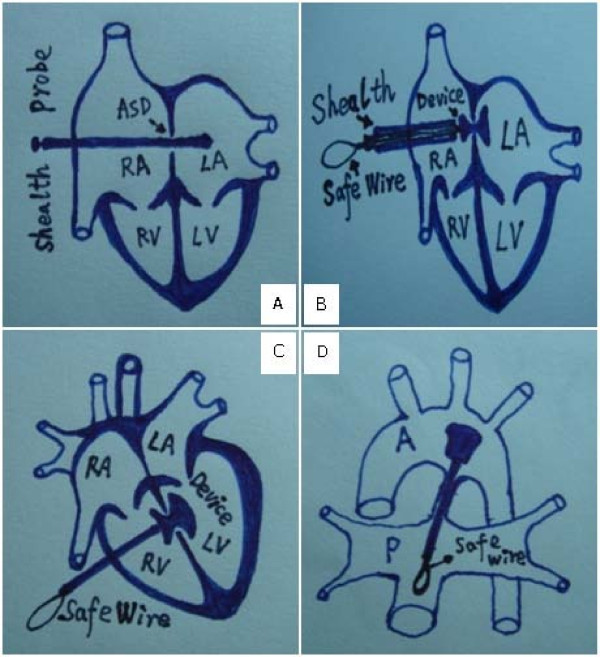
**Procedures of OPOTTMIS**. A: Through right atrium wall the outer sheath and guide probe across ASD; B: The inner sheath and the implanted and released Occluder with safe wirestraddled on the edges of ASD; C: Through the right ventricular wall the implanted and released Occluder straddled on the edges of VSD; D: Through the main pulmonary wall the implanted and released Occluder straddled on the edges of PDA; Device also referred as Occluder; Safe wire (gross silk suture) also referred as retrieval wire.

**Figure 4:**
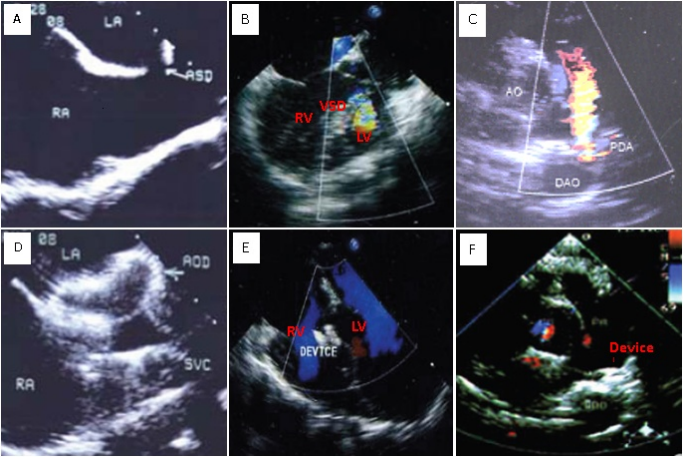
**OPOTTMIS outcomes of pre-and post-operation with TEE surveillance**. TEE images showing the abnormal blood stream disappeared post-OPOTTMIS.

The whole operating times of OPOTTMIS for simple CHD patients were approximate 20 minutes to 1 hour, and the procedures and outcomes with the TEE surveillance were shown as follows (Figure 3, Figure 4).

### (4) Announcements

①Heparin used intra-operation aims to prevent blood clotting and thrombosis and there was no protamine sulfate used after the Occluder release. Twenty-four hours after operation, a dose of 3.0 ~ 5.0 mg/kg aspirin tablet was administrated to all the patients for anticoagulation by oral once a day for about three to six months. ②The OPOTTMIS patients were asked to perform TTE and ECG checks once a month within the initial three months after operation, not to carry out intensive physical activities and hard works within the first month. Later, the patients must undertake TTE and ECG checks once every three to six months. ③if there were sever complications happened such as Occluder migration even fall off, moderate or heavy residual shunts, sever cardiac valve influence, haemolysis and thrombosis, strokes, III° AVB and infective endocarditis [5], they must be administered with the corresponding rescue treatments.

## Results

Among the consecutive 210 patients of CHD, 209 cases were performed the OPOTTMIS operation successfully, in which there were 92 cases of ASD, 63 cases of VSD and 55 cases of PDA. In the ASD groups there was 1 case of mesh-shaped ASD concomitant with persistent left superior vena cava (PLSVC) transferred to open surgery under CPB and performed atrial septum resection plus autologous pericardial patch repair and PLSVC ligation, 2 cases with mild residual shunt and 1 case with transitory II° AVB. In the VSD groups there were l case of residual shunt, 1 case of II° AVB. In addition, there were 2 patients (1 ASD and 1 VSD) with haemothorax after operation for active bleeding at the cardiac puncture sites rescued by secondary thoracic exploration and haemostatic operation. In PDA groups there were 1 case with residual shunt and 1 adult patient with moderate-heavy pulmonary hypertension died at 28 hours after operation due to pulmonary hypertension crisis.

The mortality and complication incidence of OPOTTMIS operation within 72 hours were 0.5% and 4.8%, respectively. Three days later after the operation, there was no patient death. Particularly, the complication incidences in ASD, VSD and PDA groups were 4.3% (4/92), 4.8% (3/63) and 3.6% (2/55) in sequence. Also, there were no obvious complications of Occluder migration, moderate or severe valve regurgitation, heart rupture, IE, hemolysis and thrombosis, and strokes within peri-operation.

**Figure 5:**
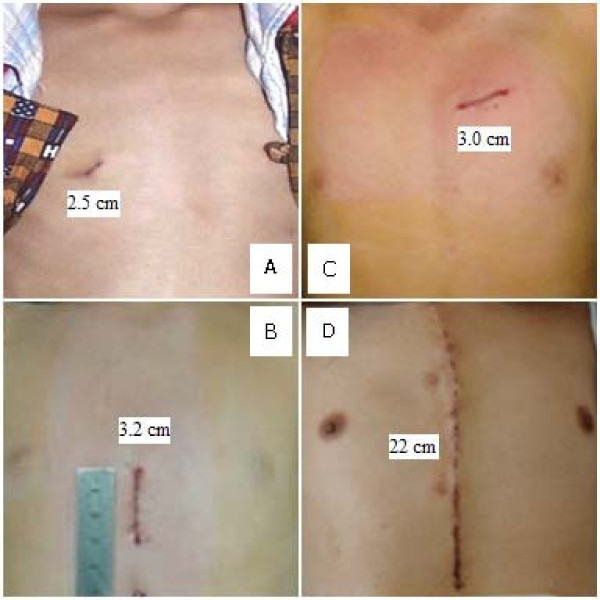
**Incisions comparison of OPOTTMIS and traditional open surgery (TOS)**. A: ASD; B: VSD; C: PDA; D: TOS.

Generally, the incisions of OPOTTMIS were 2.0 ~ 5.0 cm in length, and there were no blood transfusion and mechanical ventilation using. Their hospitalized times were 48 hours to 6 days and their total spending on OPOTTMIS were 20,000 ~ 25,000 RMB (Ren-Min-Bi, the Chinese currency). The three methods used presently for simple CHD therapy and their characteristics were compared and shown as follows (Figure 5, table 5).

**Table 5:** Characteristics comparison of TOS, MI and OPOTTMIS.

Item\Methods	TOS	P I	OPOTTMIS
Indication	wide	Relative narrow	Relative wide
Operation spot	Operating room	Catheter room	Operating room
Pathway	Open thorax	Femoral vein	Trans-thoracic MIS
Incision length	20.0 cm	0.5 ~ 1.0 cm	2 ~ 4.0 cm
CPB	Yes	No	No/Prepared
Repair ways	Transfixion/Patches	Occluder	Occluder
Operation time	> 2 h	> 2 h/2 h ±	< 1 h
X-ray radiation	No	Have	No
Injury	Heavy	Slight	Mild
Pains	Heavy	Slight	Mild
Hospitalized time	7 ~ 12 d	< 24 h	48 h ~ 6 d
Scar	Great	Almost none	Small
Cosmetic effect	weak	best	better
Spending (RMB)	20,000 ~ 35,000	40,000 ~ 45,000	20,000 ~ 25,000

TOS: traditional open surgery; PI: physician intervention; OPOTTMIS: off-pump occlusion of trans-thoracic minimal invasive surgery; RMB: Ren-Min-Bi, the Chinese currency.

All the discharged 208 patients were followed up for 3 months to 4 years by the ways of telephone contact or/and visits to outpatient department, moreover, their cardiac function were in class I ~ II according to NYH standard. Post-operation ECG and echo checks showed that there were no III° AVB, no evident Occluder migration and fall off, no moderate or severe valve regurgitation, no strokes, but for 1 VSD and 1 PDA with mild residual shunts, 2 PDA with mild hearts expansion compared to pre-operation.

## Discussion

Although traditional open surgery is a main therapy to CHD patients, it needs a large chest incision and CPB with bad cosmetic effects because of large scar, severe body injuries and many serious complications. At first, atrial septal ostomy with balloon technique attempting for palliative treatment in complex CHD may be initial sprout of Hybrid method. With Amplatzer Occluder used widely, interventional therapy to CHD patients with left to right shunts has got into new eras [26-30]. Meanwhile with improvements of the intervention equipments and operating skills the therapeutic strategy of CHD has been changed. As an integration of physician intervention and surgery techniques, hybrid approach has gradually grown into a mature operation with various advantages from an initial idea and a trial on simplex, complex or severe CHD therapy. Compared to surgery and physician intervention, hybrid approach is prompt and convenient with several advantages of applicability, maneuverability, flexibility and reciprocal to problems that cannot be settled by themselves alone, because it can reduce the risk and trauma of surgery, avoiding X-ray and catheter damages of intervention, increasing the operating efficacy, and decreasing their respective complications [31-36].

Why the devices were delivered via chest wall incisions rather than transvenous approach in the operation of OPOTTMIS? Because these incisions could supply convenient, short and straightforward operating pathways approaching to the heart puncture sites through which Occluders could reach the defects directly. Moreover, large delivery shealth and Occluders can pass through them for large defect blocking. As we knew that physician intervention occlusion on adult and large defect CHD patients may appear vascular injuries and cardiac structure damages because of angiosclerosis, pulmonary hypertension and tissue degeneration, while infants and children exposed to X-ray may cause potential marrow damages and malignant diseases. In addition, allergic patients with contrast agent are incompatible for intervention treatment. When the emergency events take place during physician intervention procedures such as Occluder migration or fall off, or the vascular and inner cardiac structures damaged or/and twisted by the long slender catheter or/and guide wire, it must be transferred to the open surgery. Also, because of the long distance and time of transportation between catheter room and operation room, the transit valuable opportunities to rescue these patients may be wasted. Now days, CPB is still indispensable to CHD therapy in most methods of MICS (minimally invasive cardiac surgery), VATS (video assisted thoracoscopic surgery) and Robotic System cardiac surgery. However, long learning curve and high cost are need to these methods so that their wide applications in the domestic are restrained [6-10].

The OPOTTMIS operation represented the humanistic and patient-oriented therapeutic spirits with short and direct pathway, without CPB interfering with physiological internal environment, avoiding potential transcatheter and guide wire injuries to the pathway vascular and cardiac inner structures such as valves, chordaes, papillary muscles, conduction blunts, etc. Also it can reduce skin scars with a favorable cosmetic outcomes, lower the spending compared to other methods, avoid X-ray damages to medical personnel and patients, as reported that X-ray may lead to chromosome and DNA damages, infertility being genitical gland injuries, even myelosuppression, leukaemia and other cancers, especially to children, adolescents and the child-bearing women [33-38].

The operating and Occluder release must be monitored with real-time ECG and TEE checks to avoid III°AVB and ensure the device at an appropriate position, which is key factor of success for OPOTTMIS [24]. Adequate inner cardiac anatomy knowledge and skilled TEE manipulating techniques are necessary for ultrasonic specialist. The relations of the delivery system, Occluder, the adjacent atrioventricular valves, coronary sinus and defect edges should be seen clearly during the operation from different planes and different angles. The release position of Occluders (especially, eccentric Occluders) must be adjusted to avoid inner cardiac structure injuries such as valves, conductive bundles, chordaes, papillary muscles and endocardium. At last, once the Occluder waist straddled on the defect edges and clamped firmly, with its umbrella lobes in an appropriate position and bearing steady strength, without residual shunts and evident influences to the adjacent inner cardiac structures, the "safe wire" stitched on the Occluder was cut off and removed.

The good advantages of OPOTTMIS [21-24] were shown as: ①Improved the security and accuracy of occlusion: Using short, large and straightforward delivery system instead of long, slender and curved sheath in physician intervention made operating procedures controlled freely. Therefore OPOTTMIS could reduce the risk of cardiac structure damages and myocardium perforations. ②Wider indications and high success rate of occlusion: OPOTTMIS was applicable to several special defects puzzling physician intervention such as large ASD (diameter > 30 mm), edge deficient ASD, lager VSD, eccentric VSD and larger PDA. Since the pathway of OPOTTMIS was short so the direction and angle of delivery systems could be controlled and regulated freely. Lager Occluder within wide sheath could supply consistent sufficient clamp strength on the defect edges, reduce device fall off and residual leakages, and improve the success rate of OPOTTMIS compared to physician intervention. ③Without vascular damages: Because its pathway doesn't go through vascular thus without vascular damages, OPOTTMIS also could be used in children with low body weight and small vascular. ④Without large chest incision and CPB using: OPPOTTMIS is minimal invasion with mild postoperative pain, fast recovery and short hospitalized time (2 - 6 days). ⑤Convenient transform to open surgery and high security: The OPOTTMIS was performed in the operating room, once emergency events happened, rescued measures or transform to open surgery could be administered immediately when needed. ⑥Short operating time: Normally, OPPOTTMIS operating times were 20 minutes to 1 hour. ⑦Excellent cosmetic effect: Small and low chest wall incisions without drainage tubes were used in OPOTTMIS with minimal dermatic scar and good cosmetic effect, especially for children and young women. ⑧Without X-ray damages. ⑨Low expenses: Generally, compared to the traditional open surgery and physician intervention methods, the spending of the OPOTTMIS was lower with a total sum of RMB 20,000 ~ 25,000 due to without blood transfusion and respiratory machine using.

## Conclusions

OPOTTMIS is a safe, feasible, effective and appropriate option for selected simple CHD (ASD, VSD and PDA) patients with good advantages of straightforward operating procedures apt to be learned and mastered, with wider indications, cosmetic incisions, mild post-operation pains, shorter hospitalized time, less hospital charges, without X-ray damages to the patients and medical staff, patient willing acceptance, and a favorable short term efficacy. However the long term outcomes and influences to heart functions should be studied in the future.

## Conflict of interests

The authors declare that they have no competing interests.

## Authors' contributions

GQK collected the clinical data and performed the statistical analysis, participated in the operation and drafted the manuscript. LZQ designed the study and performed the operation. CSF, CY and ZYH, participated in the operation. ZC was the technician of CPB when transfer to open surgery was needed. ZYL was the technician of echocardiography for intro-operation TEE surveillance. All authors read and approved the final manuscript.
